# Identifying Cardiovascular Risk by Nonlinear Heart Rate Dynamics Analysis: Translational Biomarker from Mice to Humans †

**DOI:** 10.3390/brainsci15030306

**Published:** 2025-03-14

**Authors:** Torben Hager, Agorastos Agorastos, Sven Ove Ögren, Oliver Stiedl

**Affiliations:** 1 Center for Neurogenomics and Cognitive Research, Vrije Universiteit Amsterdam, 1081 HZ Amsterdam, The Netherlands; torben.hager@gmail.com; 2 Division of Neurosciences, II. Department of Psychiatry, School of Medicine, Faculty of Health Sciences, Aristotle University of Thessaloniki, GR-54124 Thessaloniki, Greece; aagorast@auth.gr; 3 Department of Neuroscience, Karolinska Institutet, 171 77 Stockholm, Sweden; svenoveogren@yahoo.com; 4 Department of Health, Safety and Environment, Vrije Universiteit Amsterdam, 1081 BT Amsterdam, The Netherlands

**Keywords:** autonomic nervous system diseases, corticotropin-releasing factor, heart rate dynamics, neurocardiology, neuropeptide Y, neuropharmacology, parasympathetic function, sympathetic function, serotonin

## Abstract

Background: The beat-by-beat fluctuation of heart rate (HR) in its temporal sequence (HR dynamics) provides information on HR regulation by the autonomic nervous system (ANS) and its dysregulation in pathological states. Commonly, linear analyses of HR and its variability (HRV) are used to draw conclusions about pathological states despite clear statistical and translational limitations. Objective: The main aim of this study was to compare linear and nonlinear HR measures, including detrended fluctuation analysis (DFA), based on ECG recordings by radiotelemetry in C57BL/6N mice to identify pathological HR dynamics. Methods: We investigated different behavioral and a wide range of pharmacological interventions which alter ANS regulation through various peripheral and/or central mechanisms including receptors implicated in psychiatric disorders. This spectrum of interventions served as a reference system for comparison of linear and nonlinear HR measures to identify pathological states. Results: Physiological HR dynamics constitute a self-similar, scale-invariant, fractal process with persistent intrinsic long-range correlations resulting in physiological DFA scaling coefficients of α~1. Strongly altered DFA scaling coefficients (α ≠ 1) indicate pathological states of HR dynamics as elicited by (1) parasympathetic blockade, (2) parasympathetic overactivation and (3) sympathetic overactivation but not inhibition. The DFA scaling coefficients are identical in mice and humans under physiological conditions with identical pathological states by defined pharmacological interventions. Conclusions: Here, we show the importance of tonic vagal function for physiological HR dynamics in mice, as reported in humans. Unlike linear measures, DFA provides an important translational measure that reliably identifies pathological HR dynamics based on altered ANS control by pharmacological interventions. Central ANS dysregulation represents a likely mechanism of increased cardiac mortality in psychiatric disorders.

## 1. Introduction

The beat-by-beat fluctuation of heart rate (HR) is regulated by the modulation of the myogenic pacemaker systems of the heart through the parasympathetic (PNS) and the sympathetic nervous system (SNS), which constitute the autonomic nervous system (ANS). Both systems are generally interdependent to maintain blood flow in the body with proper blood pressure to support physiological and metabolic demands ranging from posture changes via physical activity to emotional challenges. Thus, internal and external sensors affect the regulation through feedback systems acting on different time scales from fast baroreflex feedback to slow endocrine changes. The complexity of cardiovascular regulation under physiological conditions is impressively presented in the systems analysis diagram created by Guyton et al. [1].

The high comorbidity of affective disorders and cardiovascular disease, particularly the elevated risk of cardiovascular failure after emotionally challenging events [2,3], indicates a crucial role of stress-induced adjustments caused by withdrawal of the parasympathetic and activation of the sympathetic–adrenal medullary system [4]. The neurocircuitry of emotions including fear [5,6] largely overlap with the central autonomic network [7,8]. Even insular cortex forebrain stimulation results in arrhythmogenesis through altered central ANS function [9]. Mutations in the human KCNQ1 gene, which encodes a cardiac and forebrain-specific delayed rectifying potassium channel, link epileptic seizures and arrhythmias to sudden unexpected death in epilepsy (SUDEP) in mice. This finding indicates the dual arrhythmogenic potential of an ion channelopathy through increased neuronal excitability in the brain and prolonged QT syndrome in the heart [10,11]. Taken together, these results underscore the need for a better understanding of the brain–heart interaction (hence neurocardiology [12]) with highly sensitive readouts to identify altered and pathological sympatho-vagal balance.

Clinically, reduced HR variability (HRV) based on linear diagnostic measures derived from the time and frequency domains [13] serves as an index of reduced regulatory capacity. Reduced HRV is considered a cardiovascular risk factor [14] associated with increased sudden cardiac death [15]. Generally, the HRV is inversely related to the absolute HR value along its physiological range and is not independent [16]. The approximate exponential relationship approaches minimum RMSSD values at maximum HR (~800 bpm) or minimal RR interval (~75 ms) in mice. Deviating slopes of RR versus RMSSD values compared to the physiological range indicate altered autonomic regulation as shown for NPY treatment [17]. However, it is unclear whether small HRV reductions are meaningful to conclude pathological states. In addition, a highly elevated HRV may also indicate a pathological-like state if based on ectopic beats and/or arrhythmias [18].

Generally, two functional properties of HR dynamics, non-stationarity and interdependence, formally prohibit the use of linear analysis [19,20]. Non-stationarity refers to the drift-like behavior of HR whereas interdependence refers to the correlation of heartbeat intervals in its temporal sequence. Nonlinear measures provide useful information on the dynamical state with superior discrimination of physiological versus pathological changes [21]. The clinical significance of nonlinear (fractal) analysis of HR dynamics in humans for the assessment of cardiovascular risk has been demonstrated in several studies [20,22]. Various nonlinear dimensionless measures of the dynamical properties of HR may serve as valuable diagnostic tools and clinical biomarkers in humans [23]. However, despite freely available software packages for nonlinear analyses (see www.physionet.org, accessed on 27 September 2024), their use is quite limited, largely due to complexity [24] and limited knowledge about the comparability of different nonlinear measures of HR dynamics. To date, a nonlinear characterization of HR dynamics based on a wider range of different pharmacological substances affecting cardiovascular regulation is available only in rats [25].

The first aim of this study was to compare linear (HR and its variability) and nonlinear HR measures (detrended fluctuation analysis, DFA) after various behavioral and pharmacological interventions. DFA, originally developed by Peng et al. [19,26], is an important tool to quantify HR time series, with dynamics far from equilibrium thereby indicating long-range heartbeat interval correlations in healthy humans which are compromised in patients with congestive heart failure. For the present study in mice, a spectrum of behavioral states was used to determine the range of physiological adjustments in untreated animals. Pharmacological interventions included drugs acting on the ANS and different central receptors that are implicated in affective disorders, e.g., depression and post-traumatic stress disorder (PTSD), since they show high comorbidity with cardiovascular risk. Euclidian clustering was used to identify similarities and differences across interventions based on linear and nonlinear measures. As a second aim, the translational relevance of nonlinear measures was assessed by comparing the HR measures obtained from mice with those from human studies with different experimental conditions. This includes an extended discussion of pathological states such as heart transplantation from the existing literature, identifying identical acute functional properties with those elicited by pharmacological interventions in mice irrespective of species-specific differences in absolute HR values.

## 2. Materials and Methods

The method spectrum applied for the conducted experiments is based on >25 years of experience in ECG recordings by radiotelemetry in freely moving (untethered) mice in combination with optimized peripheral and central injections and ECG/HR analysis, as explained below in more detail.

### 2.1. Subjects

The experiments were performed with a total of 176 male C57BL/6N mice (Charles River, Sulzfeld, Germany and ‘s-Hertogenbosch, The Netherlands) obtained at an age of 8 weeks. They were individually housed with nesting material in standard-type II Macrolon cages with free access to food and water and were kept on a 12 h dark/light cycle with lights switched on at 7 a.m. Mice were 11–13 weeks of age at the time of testing, which was performed during the light phase to minimize the effects of physical activity on cardiovascular readouts. All animal experiments were approved by the ethics committees and performed in accordance with the European Council Directive 86/609/EEC and directive 2010/63/EU at the Max Planck Institute for Experimental Medicine, Göttingen, Germany (1997–2003), and the Vrije Universiteit Amsterdam, The Netherlands (2004–2016).

To compare HR dynamics in mice with those in humans, we included data from previous human experiments with heart transplantation, congestive heart failure, during sleep and from different postures and diurnal phases, largely analyzed as described here for nonlinear approaches [27].

### 2.2. ECG Surgery, ECG Recording and Data Processing

ECG signals of mice were recorded by radiotelemetry using miniature ECG radio-transmitters (TA10EA-F20 and ETA-F10, Data Sciences International, St. Paul, MN, USA) implanted into the abdominal cavity of mice with the ECG electrodes placed subcutaneously in lead II position as described before [28,29] under general anesthesia and perioperative analgesia. Experiments were performed after full recovery, i.e., 14–21 days after surgery. All ECG recordings lasted for 18 min, providing ~10^4^ beats/mouse under physiological conditions, which roughly equals 2 h ECG recordings in humans.

The ECG signal emitted by the radio-transmitter was detected by a receiver (RLA1020, Data Sciences) and converted to an analog signal (ECG Output Adapter Option RO8, Data Sciences). This signal was digitally recorded (LabChart 7.1, PowerLab, ADInstruments, Spechbach, Germany) at a 4 kHz sampling rate and stored. The digitized ECG was analyzed offline (HRV 1.4 for LabChart, ADInstruments, Oxford, United Kingdom) to obtain discrete time points corresponding to the successive R-wave maxima. Ectopic (bradycardic) beats, typically 1 in 104 beats, were fitted by a third-order autoregressive model to the beat interval data stream using multiples of the interquartile distance as the detection threshold and were replaced by linear-spline interpolation [30,31]. This is important since incorrect RR intervals including ectopic beats (~1/10,000 beats in C57BL/6N mice [31]) can impair the quality of nonlinear DFA [32]. DFA [26] was performed as previously applied and validated by us in mice [31] and humans [20,33]. The DFA scaling value α theoretically ranges from α = 0.5, indicating absent correlations (i.e., white noise), to α~1.0, indicating long-term correlations (i.e., 1/f-noise), to α = 1.5, indicating short-term correlations (i.e., Brownian noise). The need for thorough (and time-consuming) data pre-processing by experts, as recommended by the HRV Task Force [13], is a fundamental prerequisite prior to final data analysis [23]. Lack of compliance with these standards will negatively affect the quality of results or may not provide the necessary data quality for nonlinear analyses. HR in beats per min (bpm) and the standard deviation (SD) of the NN (RR) intervals (SDNN; in ms) served as linear time-domain measures.

### 2.3. Drugs and Administration

All drugs and dosages used are provided in Table 1. These drugs, their dosages and their administration routes were selected on the basis of previous studies [17,21,29,34]. All drugs were freshly dissolved on the day of use. They were injected subcutaneously into the scruff of the neck or intracerebroventricularly into the lateral ventricles using a bilateral symmetrical brain cannula implanted 5 days prior to the experiment, as described before [17,29]. Central drug injection was necessary because the neuropeptides neuropeptide Y (NPY; 36 amino acids) and ovine corticotropin-releasing factor (oCRF; 41 amino acids) do not cross the blood–brain barrier. Drug administration was performed during a brief ~30–90 s isoflurane anesthesia period, as used by us in many studies before [17,21,29,34]. This brief anesthesia is necessary in mice as it prevents the restraining procedure required for drug injection (especially CNS infusion) from serving as a considerable stressor, causing prolonged tachycardia in addition to hyperthermia in the wake state [35]. Brief inhalation anesthesia does not affect the DFA results. During anesthesia, ECG was measured in mice placed on a heat pad at 37 °C to avoid hypothermia, which would alter ANS control, thereby affecting HR dynamics [36].

### 2.4. Experimental Conditions

Experiments were performed in the home cage of mice, except when the effects of anesthesia, novelty and restraint stress were tested. Novelty measurements occurred in a novel cage into which mice were transferred from their home cage located in an adjacent housing room [28]. For restraint stress tests, mice were immobilized, i.e., fixed with the ventral side up on the ECG receiver. Sleep was determined on the basis of minimal and only transient ECG amplitude changes during the 18 min ECG recording in the middle of the light phase when locomotor activity of C57BL/6N mice is generally absent [37]. Only mice that provided ECG data as controls during novelty, restraint and sleep were subsequently tested in a single pharmacological intervention to avoid sensitization and other potential interactions with ECG measures through repeated drug treatment.

### 2.5. Statistical Analyses

Data were analyzed by analysis of variance (ANOVA) or by Welch ANOVA in case of inhomogeneity of group variances of data, as determined by Levene’s statistic (JMP 5.0.1a and StatView 5.0.1, SAS Institute, Cary, NC, USA). An error probability of *p* < 0.05 was generally accepted as statistically significant. Due to many comparisons, the *p*-values were corrected by the minimum positive false discovery rate following a previously reported procedure [38]. The threshold was set at 5% to correct for the inflated risk of type I errors, thereby lowering the value for significance to *p* < 0.023. Hierarchical clustering dendrograms were plotted based on the Euclidian distance of either the individual linear measures HR and SDNN (weighed 1:1) or the nonlinear measures α_fast_ and α_slow_ (weighed 1:1) to compare functional consequences of different pharmacological interventions with the range of physiological HR measures during behavioral states and tests.

**Table 1:** Overview of behavioral conditions and pharmacological interventions with its doses used and sample sizes to assess heart rate dynamics in mice for a wider comparison of functional effects.

Condition */Drug (Abbreviation)	Function/Drug Action	Dose	Injection Site	*n*/Group
* Control (Co)	baseline (including saline and aCSF)	/	none, icv, ip, sc ^1^	23
* Novelty (No)	behavioral stressor	/	/	8
* Restraint (Re)	behavioral stressor	/	/	10
* Sleep (Sl)	lowest resting-state condition	/	/	11
6-OH-Dopamine (6)	noradrenergic and dopaminergic neurotoxin: peripheral sympathectomy	200 mg/kg ^2^	ip	17
Anesthesia by ketamine/xylazine (An)	NMDA receptor antagonist/α_2_ receptor agonist (on heating pad at 37 °C)	130/13 mg/kg	ip	11
Atropine (A)	mACh receptor antagonist	2 mg/kg	ip	13
Dobutamine (Do)	β_1_ receptor agonist	15 mg/kg	ip	10
DSP-4 (D)	noradrenergic neurotoxin	100 mg/kg ^3^	sc	6
Hexamethonium (H)	nACh receptor antagonist	15 mg/kg	ip	10
Isoproterenol (I)	β receptor agonist	3 mg/kg	ip	13
Phenylephrine (P)	α_1_ receptor agonist (hypertensive)	15 mg/kg	ip	8
Robinul (glycopyrrolate) (R)	peripheral mACh receptor antagonist	0.8 mg/kg	ip	12
Sodium nitroprusside (Ni)	vasodilator (antihypertensive)	0.18 mg/kg	ip	12
Sotalol (S)	peripheral β receptor antagonist	2 mg/kg	ip	12
Sotalol + Atropine (SA)	β + mACh receptor antagonists	2 mg/kg (both)	ip	11
Zatebradine (Z)	sinus node (HCN channel) inhibitor	2 mg/kg	ip	11
Ovine CRF (oC)	preferential CRF_1_ receptor agonist	210 ng/mouse	icv	12
Neuropeptide Y (N)	neuropeptide Y_1–5_ receptor agonist	500 ng/mouse	icv	10
8-OH-DPAT (8)	5-HT_1A_/5-HT_7_ receptor agonist	0.5 mg/kg	sc	8

Abbreviations: aCSF, artificial cerebrospinal fluid; CRF, corticotropin-releasing factor; HCN, hyperpolarization-activated cyclic nucleotide-gated channel; icv, intracerebroventricular; ip, intraperitoneal; mACh, muscarinic acetylcholine; nACh, nicotinic acetylcholine; NMDA, N-methyl-D-aspartate; sc, subcutaneous. * Experimental conditions without drug treatment. ^1^ Pooled data from all injection sites. ^2^ Tested 7 days after the third 6-OH-dopamine injection of 40/80/80 mg/kg given at 24 h intervals. ^3^ Tested 10 days after the second DSP-4 injection of 50 mg/kg given at 24 h intervals.

## 3. Results

### 3.1. Effects of Behavioral States and Pharmacological Interventions on Linear Heart Rate Measures

**Figure 1:**
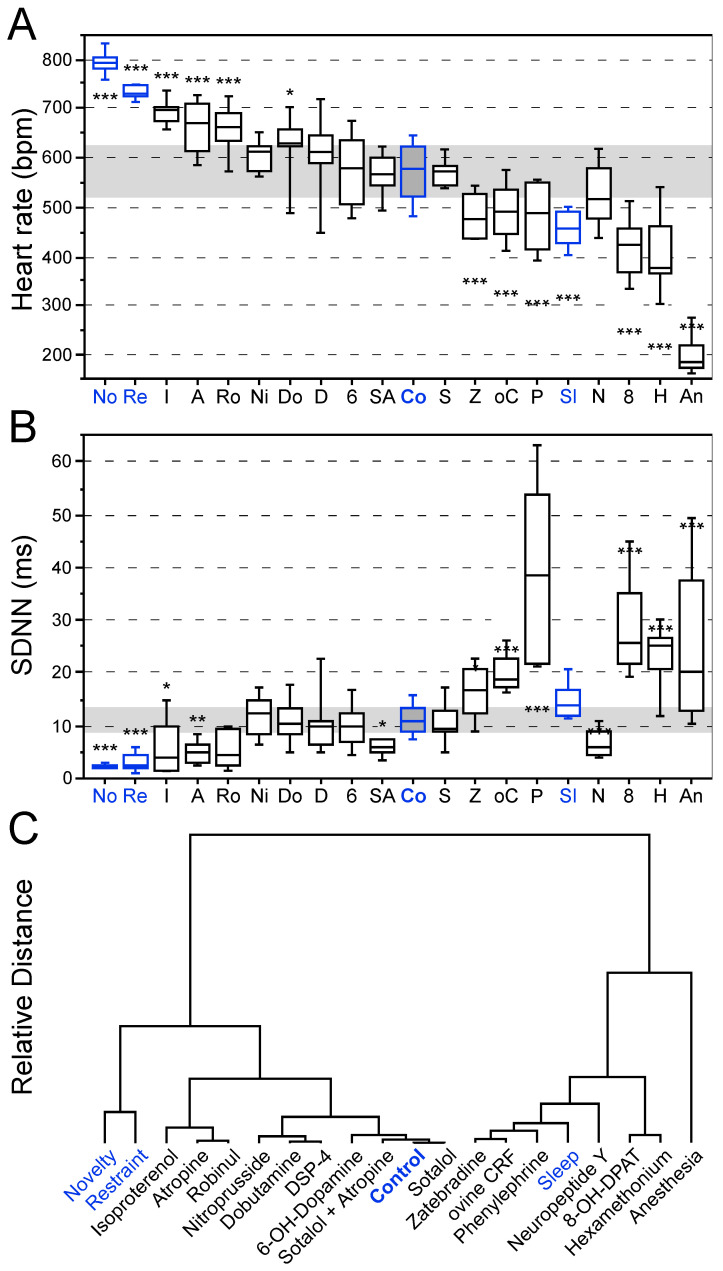
Linear heart rate measures in mice. Heart rate (**A**) and SDNN (**B**) as a function of treatment according to their Euclidian clustering (**C**). Clustering is based on the two parameters (**A**,**B**) weighed with equal contribution. Blue values and labels indicate behavioral interventions. HR (**A**) and SDNN values (**B**) indicate the generally inverse relation between these two measures. Values are based on 18 min ECG recordings in mice. Asterisks denote significant differences versus control group values: * *p* < 0.05, ** *p* < 0.01, *** *p* < 0.001.

Overall, highly significant differences were identified for HR (F_19,69.16_ = 115.82; *p* < 0.0001) and SDNN values (F_19,68.74_ = 44.04; *p* < 0.0001) between the experimental groups (Figure 1A,B). Based on the two values, cluster analysis was performed to determine the similarity of treatment effects on HR and SDNN based on a Euclidian dendrogram with identical weight for both measures (Figure 1C).

Under physiological conditions in the home cage (awake, no physical activity), the mean HR of mice was ~570 bpm and the SDNN was ~10 ms (Figure 1A,B). Novelty exposure resulted in an HR increase to maximum physiological levels (~790 bpm) and a concomitantly decreased SDNN (~2.4 ms). Restraint stress led to a lower absolute HR (~730 bpm) with slightly higher SDNN (~3.3 ms). Isoproterenol, atropine and robinul increased HR and concomitantly decreased SDNN when compared to control group values (Figure 1A,B).

Many drug treatments (e.g., nitroprusside, dobutamine, DSP-4, 6-OHDA, sotalol plus atropine, sotalol; see Table 1) did not significantly affect linear HR measures or had only mild effects (Figure 1A,B). In contrast, zatebradine, oCRF, phenylephrine, sleep, 8-OH-DPAT, hexamethonium and anesthesia generally decreased HR and increased HRV (Figure 1A,B). The most extreme bradycardia was elicited by ketamine/xylazine injection anesthesia with a median HR below 200 bpm.

Overall, the different treatments resulted in a wide range of HR values from very high (novelty) to very low (anesthesia) with SDNN values that generally were inversely related to HR (Figure 1A,B). An exception was the very low SDNN value by NPY (~6.8 ms) despite a median HR of slightly above 500 BPM. Phenylephrine (~39.4 ms), 8-OH-DPAT (~28.7 ms) and anesthesia (~25.4 ms) substantially increased SDNN values above those observed during sleep (~14.9 ms).

Euclidian cluster analysis of the linear measures (Figure 1C) separated the different interventions and experimental conditions into two subgroups. The first subgroup comprises HR values in the upper range with lower HRV from novelty exposure to sotalol including the baseline values of the control group. The second subgroup contains interventions resulting in lower HR values with generally increased HRV including anesthesia, hexamethonium, 8-OH-DPAT, NPY, phenylephrine, ovine CRF and zatebradine.

Considering the physiological range of linear HR measures during behavioral interventions and sleep, deviant measures such as substantially increased SDNN values were identified for ovine CRF, phenylephrine, 8-OH-DPAT and hexamethonium in addition to an extremely low HR by anesthesia.

### 3.2. Effects of Behavioral States and Pharmacological Interventions on Nonlinear Heart Rate Measures

**Figure 2:**
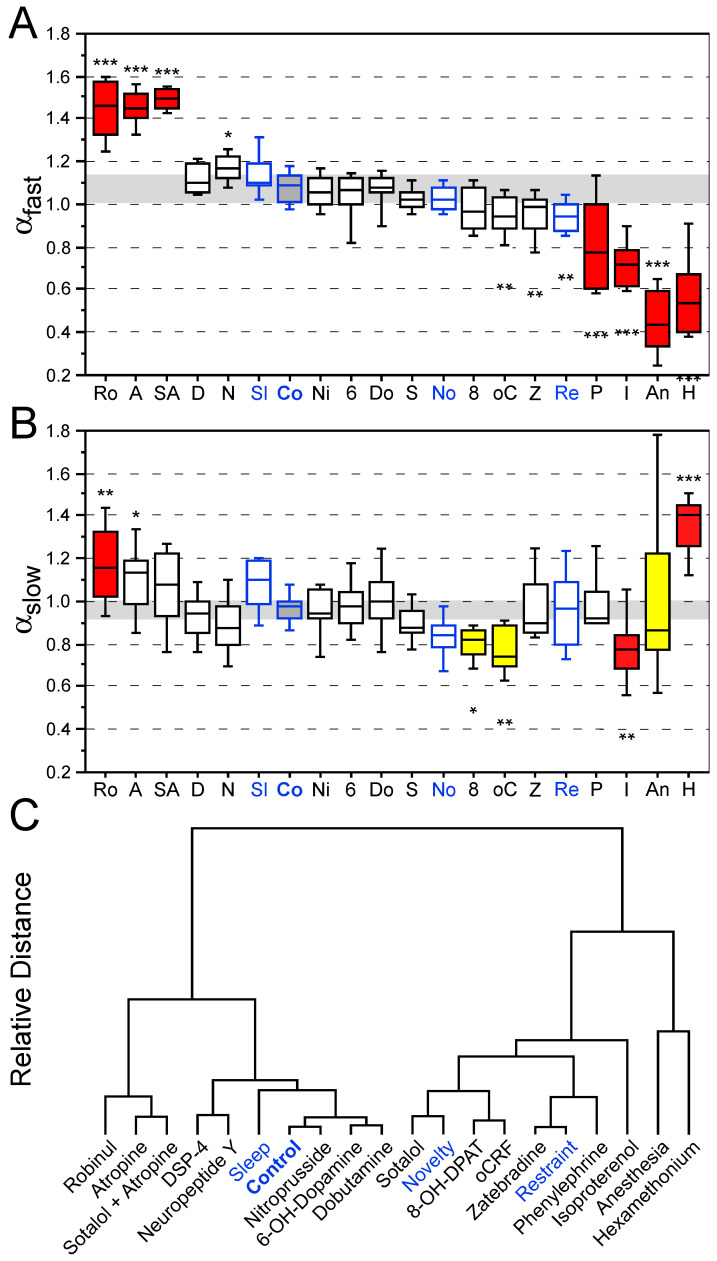
Nonlinear measures in mice. DFA measures α_fast_ (**A**) and α_slow_ (**B**) as a function of treatment according to Euclidian clustering (**C**). Clustering is based on the two parameters (**A**,**B**) weighed with equal contribution. Blue values and labels indicate behavioral interventions. Partial (yellow boxes) to complete (red boxes) decoupling of physiological regulatory systems (autonomic nervous system control) are visible at both edges of the cluster with the inner range covering physiological values. Values are based on 18 min ECG recordings in mice. Asterisks denote significant differences versus control group values: * *p* < 0.05, ** *p* < 0.01, *** *p* < 0.001.

Overall, highly significant differences were identified for α_fast_ values (F_19,69.38_ = 64.87; *p* < 0.0001) and the α_slow_ values (F_19,68.41_ = 8.43; *p* < 0.0001) between the experimental groups (Figure 2A,B). Cluster analysis was used to determine the similarity of treatment effects based on a Euclidian dendrogram with identical weight of α_fast_ and α_slow_ (Figure 2C).

Under normal physiological conditions in the home cage, the scaling coefficients α_fast_/α_slow_ were close to 1 (Figure 2A,B). The scaling coefficient was shifted towards α_fast_ = 1.5 by robinul and atropine (±sotalol), indicating the change from long-term to short-term correlation (Brownian noise). In contrast, hexamethonium, anesthesia and isoproterenol shifted the dynamical properties towards a random pattern (white noise) with α_fast_ = 0.5.

Novelty exposure resulted in reduced long-range correlation of α_slow_~0.83 (Figure 2). Restraint stress led to a lower shift in DFA values from physiological values (Figure 2). The atropine-induced shift of α_fast_ = 1.5 is indicative of a lack of parasympathetic cardiac control. The α_1/2_ agonist isoproterenol induces tachycardia along with a breakdown of short-range (α_fast_ = 0.68) and long-range correlations (α_slow_ = 0.75) due to combined sympathetic and concomitant parasympathetic (baroreflex) activation (enhanced sympatho-vagal antagonism).

Nonlinear cardiovascular measures indicated a different picture of effects. DFA was shifted towards α_fast_ = 1.5 by robinul, atropine and sotalol plus atropine, which all block the vagal system. Many interventions, from DSP-4 to novelty, did not alter α_fast_ in comparison to control group values. Ovine CRF, zatebradine and restraint stress lowered α_fast_ < 1.0. Phenylephrine and isoproterenol resulted in a drop of α_fast_~0.8. Anesthesia and hexamethonium produced a further drop of α_slow_~0.5. This pattern was complemented by a significant increase of α_slow_~1.2 by robinul, and a significant drop of α_slow_~0.8 by 8-OH-DPAT, oCRF and isoproterenol. In contrast, anesthesia resulted in an extremely amplified range of α_slow_ from 0.8 to 1.2 and hexamethonium increased α_slow_~1.4.

**Figure 3:**
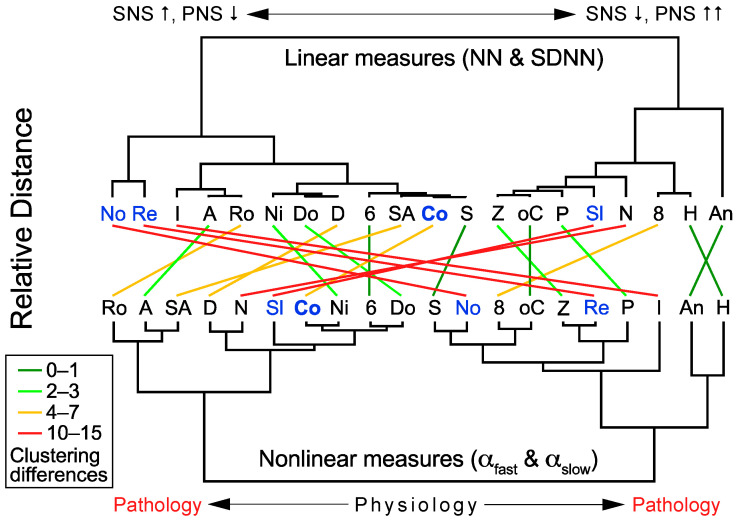
Comparison of linear and nonlinear clustering. Euclidian clustering is based on linear time-domain (NN and SDNN, as shown in Figure 1C) and nonlinear DFA measures (α_fast_ and α_slow_, as shown in Figure 2C). Clustering differences are presented between the linear measures (according to sympathetic (SNS) and parasympathetic nervous system (PNS) tone, as indicated by arrows (↑, ↓)), and nonlinear measures (from physiology to pathology as extremes), as indicated by lines between the two dendrograms. Smallest and largest positional differences are indicated by green and red lines, respectively. Pathology is characterized by PNS overactivation, which is similarly clustered by linear and nonlinear measures. In contrast, PNS inhibition by robinul (glycopyrrolate, R), atropine (A) and sotalol plus atropine (SA) is differently clustered. Additional abbreviations: 6, 6-OH-dopamine; 8, 8-OH-DPAT; An, anesthesia by ketamine/xylazine; Co, control; D, DSP-4; Do, dobutamine; H, hexamethonium; I, isoproterenol; N, neuropeptide Y; Ni, sodium nitroprusside; No, novelty; oC, ovine CRF; P, phenylephrine; Re, restraint; S, sotalol; Sl, sleep; Z, zatebradine.

Comparison of the Euclidian clustering (Figure 3) indicated substantial differences between linear time-domain (NN and SDNN) and nonlinear measures (α_fast_ and α_slow_). Conclusions on pathological HR dynamics provided solely based on linear time-domain measures are possible only for very low HR/very high HRV values (hexamethonium and anesthesia). In contrast, the DFA measures are clustered with the two outer areas (left: robinul, atropine and sotalol plus atropine; right: phenylephrine, isoproterenol, hexamethonium and anesthesia), indicating pathological HR dynamics, whereas the inner clustering area represents the physiological range of HR dynamics. Hence, the DFA provides superior sensitivity to identify pathologically altered HR dynamics in mice.

## 4. Discussion

Linear and nonlinear HR measures were analyzed in response to a wide range of behavioral and pharmacological interventions in C57BL/6N mice that have not been reported on this level before, providing novel conclusions about transiently altered pathological HR dynamics. Based on our experience also with C57BL/6J mice, we have no evidence for C57BL/6 substrain differences in cardiovascular function at younger ages of up to 5 months. However, C57BL/6J substrain differences emerge at 24 months of age [39]. 

Here, we demonstrate the dissociation of the interpretation of physiological versus pathological HR dynamics in mice because the nonlinear DFA measures identified pathological conditions (8-OH-DPAT, ovine CRF, zatebradine) despite a presumably beneficial HRV increase. The DFA measure enables a direct comparison of the dynamical properties of the heartbeat interval fluctuations of mice and humans with similar changes based on the same functional interventions. Strongly altered nonlinear DFA scaling coefficients (α ≠ 1) were indicative of pathological states of HR dynamics. These were elicited by (1) parasympathetic blockade, (2) parasympathetic overactivation and (3) sympathetic overactivation (but not inhibition), which result in parasympathetic counter-regulation (e.g., baroreflex feedback), resulting in enhanced sympatho-vagal antagonism. This study demonstrates that HR dynamics under physiological conditions are determined by ANS control depending on tonic vagal activity (neither absent nor extremely elevated) in both mice and humans, irrespective of species-specific allometric HR differences in mammals [40].

### 4.1. Linear Heart Rate Measures in Mice and Autonomic Function

The acute pharmacological interventions tested here yielded clear-cut effects on autonomic regulation and allowed us to better compare and interpret the observed nonlinear effects. The pharmacological blockade of muscarinic acetylcholine receptors by atropine underscores the importance of tonic vagal (inhibitory) control for the dynamical properties under physiological conditions as well as the changes observed under stressful conditions. Vagal blockade is mimicked by anesthesia in mice and heart transplantation in humans, resulting in functional denervation of the heart with similar functional consequences based on DFA measures across species irrespective of absolute HR values.

As in humans, HR dynamics in mice are under tonic PNS control [34,41,42]. This contradicts the claim that ‘the lack of HR increase in conscious mice after parasympathetic blockade or sympathetic stimulation further supports the supposition of predominant sympathetic activity or low vagal tone under physiological conditions’ [43]. However, this conclusion must be rejected because the reported ‘baseline’ HR of 714 bpm due to insufficient post-surgical recovery in fact represents a substantially elevated HR in C57BL/6J and C57BL/6N mice. Their maximum HR is generally ~800 bpm [31,34,44]. Only p11-deficient mice exhibit maximum HR values of up to 850 bpm [45]. In contrast, resting-state HR in C57BL/6J and C57BL/6N mice normally ranges from 500 to 600 bpm [34,41]. The atropine-induced HR increase parallels the well-known HR increase in human heart transplant recipients. This HR increase indicates tonic PNS function for lower HR under resting-state conditions with similar dynamical consequences, i.e., absent short-range correlation in mice and humans (see Section 4.2).

The role of the sympathetic system under basal stress-free conditions is low. Sympathetic inhibition by the peripheral β antagonist sotalol exerted no effects on HR dynamics. Noradrenergic depletion by 8-OH-dopamine and DSP-4 both did not alter the dynamical properties in rats [46]. However, sympathetic overstimulation by phenylephrine and isoproterenol affected HR dynamics in part due to baroreflex activation to counteract high blood pressure by slowing HR through vagal activation, as particularly indicated by the phenylephrine-mediated high SDNN values [41,42].

The experimental conditions determine the basal behavioral/emotional state which in turn affects HR dynamics of mice. This is evident from any short-lasting (>10 min) experiments involving handling, since HR in mice easily increases to values in the range of 750–800 bpm due to enhanced sympathetic activation and parasympathetic withdrawal [44,47,48]. Thus, conventional short-lasting tests are unsuited to assess baseline HR dynamics in mice since novelty, which increases locomotor activity [28], and emotional states, such as fear and anxiety, substantially elevated basal HR [34,45], as shown here again. The high sensitivity to fluctuating environmental conditions in mice may partly be due to limited experience because of commonly ‘impoverished’ housing conditions [44]. Therefore, home cage-based experiments or tests in habituated environments are essential for studies in mice to determine autonomic response adjustment to defined stressors without unspecific confounding effects of novelty in combination with handling [34,37,49].

Hyper- and hypo-responsiveness based on HR may serve as an indicator for altered autonomic function in response to emotional challenge secondary to differences in basal function [17,21]. Challenge-induced response differences without baseline differences can be mediated by the serotonin transporter-linked polymorphic region [50] and long-term SSRI treatment in healthy controls [51]. Altered cardiovascular responsiveness to stressors may indicate compromised adaptive (maladaptive) consequences [52]. However, personality-dependent differences in humans with regard to stress susceptibility and HRV exist [53]. Similarly, a study in isogenic C57BL/6J mice shows consistent differences in inter-individual fear response magnitudes based on HR responses to three unconditioned stressors suggesting personality trait-like differences [54], as reported on the behavioral level [37] related to epigenetic effects. Unlike the assumption that altered autonomic responsiveness may be reflected only in the response to challenging conditions [55], nonlinear methods increase the sensitivity to detect blunted tonic parasympathetic activity during the sleep phase, as recently shown in post-traumatic stress disorder [33].

Sleep in individually housed mice results in a strong HR decrease and SDNN increase without a significant change in DFA scaling coefficients [31], which differs from results in humans. Here, sleep increases PNS activity, causing a significant α decrease (Table 2; [27,56]). The unaltered HR dynamics in mice during sleep are attributed to the substantially higher basal metabolic needs to maintain body temperature than in humans [57]. Enhanced PNS activity with lower baseline values occurs during sleep in group-housed mice [58].

Despite functional similarities between mice and humans, substantial species-specific differences exist in linear cardiovascular measures. These make it difficult to clearly define pathological states without a validated reference system.

### 4.2. Nonlinear Heart Rate Dynamics in Mice to Assess Pathological States

Nonlinear methods are powerful tools to study any kind of oscillatory measure, as commonly observed in time-series analysis of physiological processes. The DFA results demonstrate the role of the parasympathetic system for short-term correlations that is shifted towards Brownian noise by atropine (α_fast_ = 1.5). Thus, the qualitative assessment of HR dynamics is favored by nonlinear analyses, because linear measures lack a clear indication of health versus disease state, e.g., as indicated by the mild HR increase and SDNN reduction in the peripherally acting muscarinic acetylcholine receptor blockade by robinul [59] but strongly impaired HR dynamics. The dose of zatebradine of 2 mg/kg, as used here, is below that described to induce arrhythmias [60] yet still indicated pathological HR dynamics, suggesting improved sensitivity to detect the threshold dose for the changeover from physiological to pathological HR dynamics undetected by linear HRV measures.

Besides novelty with HR close to its maximum, highly aversive behavioral conditions such as restraint stress elevated HR to values above 700 bpm. However, here, HR dynamics based on α_fast_ decreased significantly due to the withdrawal of the parasympathetic tone and enhanced sympathetic tone. The profound blood pressure increase observed during restraint stress [44], but not novelty exposure, very likely contributes to baroreflex feedback, lowering the maximum HR in a similar way to that observed during isoproterenol treatment in a deviant physiological state. These results show that linear HR measures are less informative than nonlinear measures in characterizing and better discriminating between these two autonomic states.

The general view that decreased HRV, as an index of attenuated parasympathetic and/or increased sympathetic function, is a predictor of increased cardiac risk [14,61,62] cannot be unequivocally supported by our data. Decreased HRV by pharmacological interventions (e.g., NPY, sotalol) did not result in pathological HR dynamics. In contrast, substantially increased HRV indicated partially dysregulated ANS control of HR dynamics. This state was induced by ovine CRF, resulting in central CRF_1_ receptor activation [29], and by 8-OH-DPAT, resulting in 5-HT_1A_ receptor-specific activation. This is blocked by a selective 5-HT_1A_ receptor antagonist, WAY-100635 [21], whereas the 5-HT_7_ receptor is not involved [63]. The adverse consequences of acute postsynaptic 5-HT_1A_ receptor stimulation by 8-OH-DPAT on HR dynamics with reduced HR associated with substantially increased HRV in mice [21] mimic the pathological symptoms of selective serotonin reuptake inhibitors (SSRIs) at high doses, as observed in humans [64,65]. This includes PQ interval prolongation (Torsade de Pointes), as indicated by a U.S. Food and Drug Administration Safety Communication on Celexa in 2013 (see http://www.fda.gov/drugs/drugsafety/ucm297391.htm, accessed on 1 October 2024). Thus, abnormal states of HR dynamics include overactivation of both ANS branches, referred to as enhanced sympatho-vagal antagonism [10,18,28], despite seemingly beneficial HRV increase demanding thorough ECG subinterval analyses to identify pathological alterations such as PQ prolongation.

### 4.3. Heart Rate Dynamics in Humans—Translational Validity

HR dynamics in awake humans provide DFA values close to 1 (Table 2) irrespective of their state. During the nighttime, a drop in the scaling coefficient occurs (α = 0.86), which is attributed to higher vagal tone during sleep. This higher vagal tone during sleep is blunted in people with PTSD [33]. However, the overall 24 h values were similar to daytime values. The scaling coefficient was lower in children (α = 0.75) than in adults due to not yet fully matured PNS [27], a developmental process that is still incompletely understood [66]. Pathological HR dynamics are identified in a group of congestive heart failure patients. The group-averaged DFA exponent α = 1.24 differed significantly from α~1, as determined in normal healthy subjects, and moved towards more short-term correlation (Table 2).

A straightforward approach addressing the significance of a lack of autonomic cardiac control for the fractal dynamics of HR are studies in recipients of a cardiac transplant. The DFA value of α = 1.5 in heart transplant recipients assessed less than 2 years after transplantation indicates that the denervated human heart exhibited dynamics paralleling Brownian noise (α~1.5), i.e., short-term correlation. In contrast, DFA values approached α~1.1 more than 2 years after transplantation, suggesting functional re-innervation [67]. This result is more recently supported by a more recent 10-year longitudinal study [68]. The functional consequence of denervation can be mimicked by atropine treatment and anesthesia in mice. The importance of tonic PNS activity for the dynamical (fractal) properties has also been shown by atropine treatment in humans [69]. These data indicate the same functional principles across different species underlying the fully translational DFA measure. These findings underscore the importance of efferent brain–heart connection besides afferent heart–brain connections from peripheral sensors for interoceptive awareness. Abnormal functional connectivity of afferents is implicated in anxiety disorders [70].

**Figure 4:**
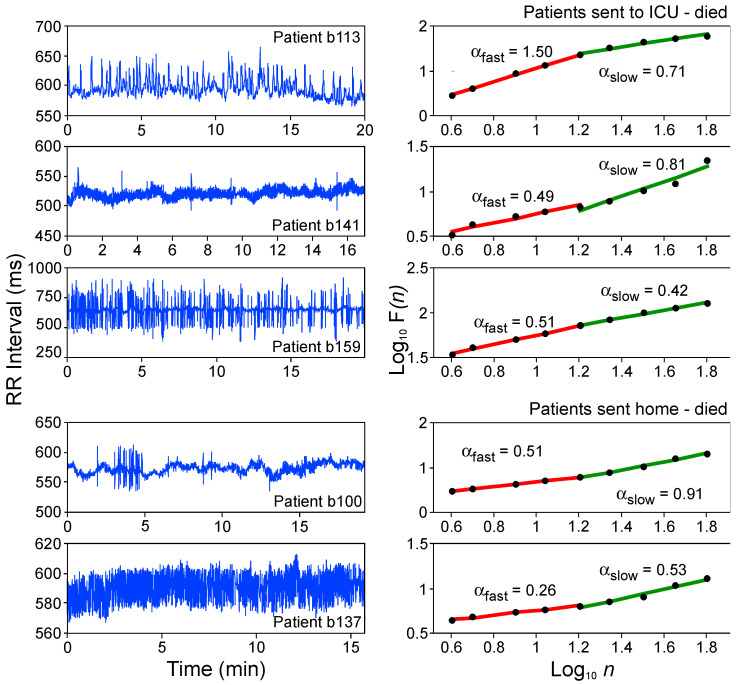
Examples of 5 heartbeat interval time series (note the different *Y*-axis ranges; (**left panels**)), and corresponding DFA scaling coefficients (**right panels**) of five patients evaluated for potential cardiac infarction due to acute chest pain and who did not display any ECG alterations (no sign of infarction). These 5 out of 323 patients were identified in a blinded study as high-risk patients for cardiac death based on the strong deviation of DFA coefficients for α_slow_ and/or α_fast_ from the physiological value of α~1. Three patients (b113, b141 and b159) were admitted to the intensive care unit (ICU). Two patients (b100 and b137) were sent home with telemetric ECG recording but in retrospect should have been admitted to the hospital (modified from [27]).

To potentially identify patients at high risk for lethal outcome in the absence of pathological ECG pattern alterations, DFA was applied to ECG data of a group of 323 patients admitted to the hospital for an evaluation of acute chest pain. In DFA plots based on 15 min ECG recordings, different α_slow_ and/or α_fast_ f slopes were observed (Figure 4).

Out of a total of 323 patients, 13 died for cardiac reasons within a period of 3 months. Furthermore, nine patients, on admission to intensive care, were identified as high-risk candidates for cardiac death based on highly abnormal HR dynamics with scaling properties approaching white noise (α = 0.5) or Brownian noise (α = 1.5). Hence, DFA serves as a sensitive diagnostic method to identify risk for cardiac mortality in humans solely based on altered HR dynamics in the absence of pathological ECG patterns or prior history of cardiovascular disease.

These results underscore the translational properties of DFA scaling values independent of species-specific HR differences in mice and humans. Furthermore, normothermic pigs exhibited HR dynamics of α~1, whereas hypothermia (28 °C) resulted in HR dynamics of α~1.5 [27], signifying the loss of parasympathetic tone. This effect is similar to that observed in heart transplant recipients within the first two years after transplantation [27]. Tonic PNS function is known in humans [22], rats [25] and rabbits [71], pointing towards a more general principle in endothermic mammalian species under physiological baseline conditions, i.e., without exposure to stressors and during normothermia.

Beyond unifractal measures such as DFA scaling coefficients used here, nonlinear methods (reviewed in [23]) offer a wide analytical spectrum including multifractal measures [20,22,72,73] to detect altered HR dynamics with high sensitivity and high translational power. Nonlinear approaches are also superior to linear measures to detect hypoxia-induced functional changes in fetal ewes’ HRV in Langendorff preparations [73].

### 4.4. Brain–Heart Network Underlying Nonlinear Heart Rate Dynamics in Health and Disease

We here demonstrated the crucial role of tonic parasympathetic function for the long-range correlation underlying the temporal sequence of HR interval fluctuations (HR dynamics) in mice, as shown for humans, as an important index of the physiological state of brain–heart interaction [74]. A wide spectrum of different interventions was used here in mice and is required for the comparison of functional consequences. Only this allows for an optimal interpretation of drug-mediated effects on autonomic regulation when determined by less commonly used nonlinear HR measures. We provide evidence for superior functional, i.e., physiological vs. pathological identification of altered HR dynamics by nonlinear analysis compared to linear time-domain measures. Our results support earlier claims on ‘when the mean is meaningless’ because standard linear HRV measures ignore the temporal dynamics (sequence) of heartbeat interval fluctuations [20] which are interdependent but not random (as prerequisite for linear analysis). These functional properties are not restricted to modulation by cognition and emotion (mood) [75]. Altered para-sympathetic function, commonly blunted vagal tone, is a major cardiovascular risk factor [76,77] in particular association with affective disorders such as anxiety disorders [78], major depression [79] and PTSD [33,80]. Since DFA scaling can predict cardiovascular risk in the absence of ECG alterations in humans [27], altered ANS control in the absence of genuine heart disease is a likely mechanism for cardiovascular risk in these patients. Consequently, besides improved diagnostics, safety pharmacology could substantially be improved by determining the dose range of novel drugs without adverse effects on HR dynamics. Unfortunately, sensitive nonlinear analyses in preclinical in vivo screening methods and safety pharmacology are still largely lacking. However, innovative in vitro/in silico approaches for drug screening have recently introduced nonlinear complexity measures [81]. Similarly, rather than relying only on linear measures in anesthesiology [82], a better characterization of different anesthetics and their doses on cardiovascular dynamics is necessary, as recently introduced [83]. The underlying mechanisms and involved brain areas for autonomic dysregulation are largely unknown but include epileptic-like neuronal activity altering ANS function [84,85]. A better mechanistic understanding is required for the functional coupling of the forebrain with the heart via the central autonomic network including feedback connections and its output systems. Experimentally this can be achieved in animal models by optogenetic interventions [86] combined with EEG/ECG recordings [11]. This methodological approach allows for high spatio-temporal interference with brain areas to functionally characterize the neural circuits involved in central tonic parasympathetic regulation and dysregulation. Parasympathetic connectivity of the ANS with the prefrontal cortex is well described [7]. Impaired rational control of emotionality, presumably arising from prefrontal cortex hypofunction [87,88], is implicated in psychopathology. Whether prefrontal cortex hypofunction [89] may result in overall lower parasympathetic function or in lower control of emotional responsiveness including ANS regulation still requires experimental evidence.

## 5. Conclusions

In conclusion, assessment of HR dynamics by nonlinear methods serves as a sensitive readout of normal brain function and indicates the importance of the parasympathetic system in the physiological state (health) of mice and humans. Assessment of HR dynamics by nonlinear measures is superior to linear measures in identifying pathological states (disease-like states) as mimicked by various pharmacological interventions. This approach can help identify the mechanisms underlying the comorbidity of affective disorders (e.g., PTSD and depression) with cardiovascular disease for refined/novel therapeutic strategies but also potential therapeutic side effects. Nonlinear measures can support studies on transient brain manipulations on a fast time scale such as optogenetic approaches for brain-area-specific investigation of the central autonomic network [7,90]. Investigations in anesthetized animals or from isolated heart experiments in vitro cannot provide data on physiological HR dynamics. Only when nonlinear measures of HR dynamics are used under physiological in vivo conditions can mouse models aid to substantially improve our understanding of the role of specific receptors in specific brain areas directly or indirectly coupled with the central autonomic network [23,91] in health and disease. The shift from linear to nonlinear methods as analytical gold standards to quantify the properties of many physiological measures including HR dynamics is more than overdue.

## Figures and Tables

**Table 2:** Human heart rate dynamics under various conditions in health and disease ^1^.

Condition/Treatment (Remarks)		*n*/Group	DFA α ^2^ ± SD
Healthy adults (unsteady state, daytime)		17	1.01 ± 0.11
Healthy adults (steady state, 24 h)		14	0.99 ± 0.07
Healthy adults (unsteady state, 24 h)		9	0.99 ± 0.04
Healthy adults (4 h daytime)		9	0.96 ± 0.05
Healthy adults (4 h nighttime)		9	0.86 ± 0.04
Healthy children (before maturation)		9	0.75 ± 0.08
Congestive heart failure (unsteady state)		20	1.24 ± 0.16
Heart transplantation (steady state, supine)	<2 years after transplantation	13	1.48 ± 0.11
	>2 years after transplantation	17	1.02 ± 0.16

^1^ Modified from [27]; ^2^ scaling coefficient α without separation into α_fast_ and α_slow_.

## Data Availability

The data that support the findings of this study are available from the corresponding author, O.S., upon reasonable request.

## References

[1] Guyton A.C., Coleman T.G., Granger H.J. (1972). Circulation: Overall regulation. Annu. Rev. Physiol..

[2] Leor J., Poole W.K., Kloner R.A. (1996). Sudden cardiac death triggered by an earthquake. N. Engl. J. Med..

[3] Steptoe A., Brydon L. (2008). Emotional triggering of cardiac events. Neurosci. Biobehav. Rev..

[4] Kvetnansky R., Sabban E.L., Palkovits M. (2009). Catecholaminergic systems in stress: Structural and molecular genetic approaches. Physiol. Rev..

[5] Hartley C.A., Phelps E.A. (2010). Changing fear: The neurocircuitry of emotion regulation. Neuropsychopharmacology.

[6] Maren S. (2011). Seeking a spotless mind: Extinction, deconsolidation, and erasure of fear memory. Neuron.

[7] Ter Horst G.J., Hautvast R.W., de Jongste M.J., Korf J. (1996). Neuroanatomy of cardiac activity-regulating circuitry: A transneuronal retrograde viral labelling study in the rat. Eur. J. Neurosci..

[8] Saper C.B. (2002). The central autonomic nervous system: Conscious visceral perception and autonomic pattern generation. Annu. Rev. Neurosci..

[9] Oppenheimer S.M., Wilson J.X., Guiraudon C., Cechetto D.F. (1991). Insular cortex stimulation produces lethal cardiac arrhythmias: A mechanism of sudden cardiac death. Brain Res..

[10] Goldman A.M., Glasscock E., Yoo J., Chen T.T., Klassen T.L., Noebels J.L. (2009). Arrhythmia in heart and brain: KCNQ1 mutations link epilepsy and sudden unexplained death. Sci. Transl. Med..

[11] Bauer J., Devinsky O., Rothermel M., Koch H. (2023). Autonomic dysfunction in epilepsy mouse models with implications for SUDEP research. Front. Neurol..

[12] Jänig W. (2016). Neurocardiology: A neurobiologist’s perspective. J. Physiol..

[13] Camm A.J., Malik M., Bigger T.J., Breithardt G., Cerutti S., Cohen R.J., Coumel P., Fallen E.L., Kennedy H.L., Kleiger R.E. (1996). Heart rate variability—Standards of measurement, physiological interpretation, and clinical use. Circulation.

[14] Shaffer F., McCraty C., Zerr C.L. (2014). A healthy heart is not a metronome: An integrative review of the heart’s anatomy and heart rate variability. Front. Psychol..

[15] Sessa F., Anna V., Messina G., Cibelli G., Monda V., Marsala G., Ruberto M., Biondi A., Cascio O., Bertozzi G. (2018). Heart rate variability as predictive factor for sudden cardiac death. Aging.

[16] De Geus E.J.C., Gianaros P.J., Brindle R.C., Jennings J.R., Berntson G.G. (2019). Should heart rate variability be “corrected” for heart rate? Biological, quantitative, and interpretive considerations. Psychophysiology.

[17] Tovote P., Meyer M., Beck-Sickinger A.G., von Hörsten S., Ögren S.O., Spiess J., Stiedl O. (2004). Central NPY-mediated alteration of heart rate dynamics in mice during expression of fear conditioned to an auditory cue. Regul. Pept..

[18] Stein P., Domitrovich P., Gottdiener J. (2005). Sometimes higher heart rate variability is not better heart rate variability: Results of graphical and nonlinear analyses. J. Cardiovasc. Electrophysiol..

[19] Peng C.-K., Mietus J., Hausdorff J.M., Havlin S., Stanley H.E., Goldberger A.L. (1993). Long-range anticorrelations and non-Gaussian behavior of the heartbeat. Phys. Rev. Lett..

[20] Meyer M., Stiedl O. (2003). Self-affine fractal variability of human heartbeat interval dynamics in health and disease. Eur. J. Appl. Physiol..

[21] Youn J., Hager T., Misane I., Pieneman A.W., Jansen R.F., Ögren S.O., Meyer M., Stiedl O. (2013). Central 5-HT_1A_ receptor-mediated modulation of heart rate dynamics and its adjustment by conditioned and unconditioned fear in mice. Br. J. Pharmacol..

[22] Goldberger A.L., Amaral L.A.N., Hausdorff J.M., Ivanov P.C., Peng C.-K., Stanley H.E. (2002). Fractal dynamics in physiology: Alterations with disease and aging. Proc. Natl. Acad. Sci. USA.

[23] Agorastos A., Mansueto A.C., Hager T., Pappi E., Gardikioti A., Stiedl O. (2023). Heart rate variability as a translational dynamic biomarker of altered autonomic function in health and psychiatric disease. Biomedicines.

[24] Aubert A.E., Vandeput S., Beckers F., Liu J., Verheyden B., van Huffel S. (2009). Complexity of cardiovascular regulation in small animals. Philos. Trans. R. Soc. A.

[25] Beckers F., Verheyden B., Ramaekers D., Swynghedauw B., Aubert A.E. (2006). Effects of autonomic blockade on non-linear cardiovascular variability indices in rats. Clin. Exp. Pharmacol. Physiol..

[26] Peng C.-K., Havlin S., Stanley H.E., Goldberger A.L. (1995). Quantification of scaling exponents and crossover phenomena in nonstationary heartbeat time series. Chaos.

[27] Meyer M., Losa G.A., Merlini D., Nonnenmacher T.F., Weibel E.R. (2002). Fractal scaling of heartrate dynamics in health and disease. Fractals in Biology and Medicine.

[28] Tovote P., Meyer M., Ronnenberg A., Ögren S.O., Spiess J., Stiedl O. (2005). Heart rate dynamics and behavioral responses during acute emotional challenge in corticotropin-releasing factor receptor 1-deficient and corticotropin-releasing factor-overexpressing mice. Neuroscience.

[29] Stiedl O., Meyer M., Jahn O., Ögren S.O., Spiess J. (2005). Corticotropin-releasing factor receptor 1 and central heart rate regulation in mice during expression of conditioned fear. J. Pharmacol. Exp. Ther..

[30] Meyer M., Stiedl O. (2006). Fractal rigidity by enhanced sympatho-vagal antagonism in heartbeat interval dynamics elicited by central application of corticotropin-releasing factor in mice. J. Math. Biol..

[31] Stiedl O., Meyer M. (2003). Fractal dynamics in circadian cardiac time series of corticotropin-releasing factor receptor subtype-2 deficient mice. J. Math. Biol..

[32] Tarkiainen T.H., Kuusela T.A., Tahvanainen K.U.O., Hartikainen J.E.K., Tiittanen P., Timonen K.L., Vanninen E.J. (2007). Comparison of methods for editing of ectopic beats in measurements of short-term non-linear heart rate dynamics. Clin. Physiol. Funct. Imaging.

[33] Agorastos A., Boel J.A., Heppner P.S., Hager T., Moeller-Bertram T., Haji U., Motazedi A., Yanagi M.A., Baker D.G., Stiedl O. (2013). Diminished vagal activity and blunted diurnal variation of heart rate dynamics in posttraumatic stress disorder. Stress.

[34] Stiedl O., Jansen R.F., Pieneman A.W., Ögren S.O., Meyer M. (2009). Assessing aversive emotional states through the heart in mice: Implications for cardiovascular dysregulation in affective disorders. Neurosci. Biobehav. Rev..

[35] Olivier B., Zethof T., Pattij T., van Boogaert M., van Oorschot R., Leahy C., Oosting R., Bouwknecht A., Veening J., van der Gugten J. (2003). Stress-induced hyperthermia and anxiety: Pharmacological validation. Eur. J. Pharmacol..

[36] Mertens A., Stiedl O., Steinlechner S., Meyer M. (2008). Cardiac dynamics during daily torpor in the Djungarian hamster, *Phodopus sungorus*. Am. J. Physiol.-Regul. Integr. Comp. Physiol..

[37] Hager T., Jansen R.F., Pieneman A.W., Manivannan S.N., Golani I., van der Sluis S., Smit A.B., Verhage M., Stiedl O. (2014). Display of individuality in avoidance behavior and risk assessment of inbred mice. Front. Behav. Neurosci..

[38] Verhoeven K.J.F., Simonsen K.L., McIntyre L.M. (2005). Implementing false discovery rate control: Increasing your power. Oikos.

[39] Walter S., Baumgarten P., Hegemann N., Häseli S.P., Deubel S., Jelleschitz J., Höhn A., Berndt N., Kuebler W.M., Grune J. (2025). Comparative phenotyping of C57BL/6J substrains reveals distinctive patterns of cardiac aging. GeroScience.

[40] He R.S., De Ruiter S., Westover T., Somarelli J.A., Blawas A.M., Dayanidhi D.L., Singh A., Steves B., Driesinga S., Halsey L.G. (2023). Allometric scaling of metabolic rate and cardiorespiratory variables in aquatic and terrestrial mammals. Physiol. Rep..

[41] Uechi M., Asai K., Osaka M., Smith A., Sato N., Wagner T.E., Ishikawa Y., Hayakawa H., Vatner D.E., Shannon R.P. (1998). Depressed heart rate variability and arterial baroreflex in conscious transgenic mice with overexpression of cardiac Gsα. Circ. Res..

[42] Just A., Faulhaber J., Ehmke H. (2000). Autonomic cardiovascular control in conscious mice. Am. J. Physiol.-Regul. Integr. Comp. Physiol..

[43] Gehrmann J., Hammer P.E., Maguire C.T., Wakimoto H., Triedman J.K., Berul C.I. (2000). Phenotypic screening for heart rate variability in the mouse. Am. J. Physiol.-Heart Circ. Physiol..

[44] Stiedl O., Tovote P., Ögren S.O., Meyer M. (2004). Behavioral and autonomic dynamics during contextual fear conditioning in mice. Auton. Neurosci. Basic Clin..

[45] Sousa V.C., Mantas I., Stroth N., Hager T., Pereira M., Jiang H., Jabre S., Paslawski W., Stiedl O., Svenningsson P. (2021). P11 deficiency increases stress reactivity along with HPA axis and autonomic hyperresponsiveness. Mol. Psychiatry.

[46] Kawamura H., Mitsubayashi H., Miao T., Shimizu T. (2005). Short and long term analysis of heart rate variations in spontaneously hypertensive rats: Effects of DSP-4 administration. Biomed. Pharmacother..

[47] Gross V., Luft F.C. (2003). Exercising restraint in measuring blood pressure in conscious mice. Hypertension.

[48] Van Bogaert M.J.V., Groenink L., Oosting R.S., Westphal K.G.C., Van Der Gugten J., Olivier B. (2006). Mouse strain differences in autonomic responses to stress. Genes Brain Behav..

[49] Voikar V., Gaburro S. (2020). Three pillars of automated home-cage phenotyping of mice: Novel findings, refinement, and reproducibility based on literature and experience. Front. Behav. Neurosci..

[50] Agorastos A., Kellner M., Stiedl O., Muhtz C., Becktepe J.S., Wiedemann K., Demiralay C. (2014). The 5-HTTLPR genotype modulates heart rate variability and its adjustment by pharmacological panic challenge in healthy men. J. Psychiatr. Res..

[51] Agorastos A., Kellner M., Stiedl O., Muhtz C., Wiedemann K., Demiralay C. (2015). Blunted autonomic reactivity to pharmacological panic challenge under long-term escitalopram treatment in healthy men. Int. J. Neuropsychopharmacol..

[52] Koolhaas J.M., Bartolomucci A., Buwalda B., de Boer S.F., Flügge G., Korte M., Meerlo P., Murison R., Olivier B., Palanza P. (2011). Stress revisited: A critical evaluation of the stress concept. Neurosci. Biobehav. Rev..

[53] Zohar A.H., Cloninger C.R., McCraty R. (2013). Personality and heart rate variability: Exploring pathways from personality to cardiac coherence and health. Open J. Soc. Sci..

[54] Liu L., Wei L., Kuang J., Tsien J.Z., Zhao F. (2014). Heart rate and heart rate variability assessment identifies individual differences in fear response magnitudes to earthquake, free fall, and air puff in mice. PLoS ONE.

[55] Berntson G.G., Sarter M., Cacioppo J.T. (1998). Anxiety and the cardiovascular reactivity: The basal forebrain cholinergic link. Behav. Brain Res..

[56] Penzel T., Kantelhardt J.W., Grote L., Peter J.-H., Bunde A. (2003). Comparison of detrended fluctuation analysis and spectral analysis for heart rate variability in sleep and sleep apnea. IEEE Trans. Biomed. Eng..

[57] White C.R., Seymour R.S. (2005). Allometric scaling of mammalian metabolism. J. Exp. Biol..

[58] Späni D., Arras M., König B., Rülicke T. (2003). Higher heart rate of laboratory mice housed individually vs in pairs. Lab Anim..

[59] Holschneider D.P., Scremin O.U., Chialvo D.R., Chen K., Shih J.C. (2002). Heart rate dynamics in monoamine oxidase-A- and -B-deficient mice. Am. J. Physiol.-Heart Circ. Physiol..

[60] Stieber J., Wieland K., Stöckl G., Ludwig A., Hofmann F. (2006). Bradycardic and proarrhythmic properties of sinus node inhibitors. Mol. Pharmacol..

[61] Stein P.K., Kleiger R.E. (1999). Insights from the study of heart rate variability. Annu. Rev. Med..

[62] Huikuri H.V., Stein P.K. (2013). Heart rate variability in risk stratification of cardiac patients. Prog. Cardiovasc. Dis..

[63] Eriksson T.E., Holst S., Stan T.L., Hager T., Sjögren B., Ögren S.O., Svenningsson P., Stiedl O. (2012). 5-HT1A and 5-HT7 receptor crosstalk in the regulation of emotional memory: Implications for effects of selective serotonin reuptake inhibitors. Neuropharmacology.

[64] Zareba W. (2007). Drug induced QT prolongation. Cardiol. J..

[65] Unterecker S., Warrings B., Deckert J., Pfuhlmann B. (2012). Correlation of QTc interval prolongation and serum level of citalopram after intoxication—A case report. Pharmacopsychiatry.

[66] Harteveld L.M., Nederend I., ten Harkel A.D.J., Schutte N.M., de Rooij S.R., Vrijkotte T.G.M., Oldenhof H., Popma A., Jansen L.M.C., Suurland J. (2021). Maturation of the cardiac autonomic nervous system activity in children and adolescents. J. Am. Heart Assoc..

[67] Meyer M., Marconi C., Ferretti G., Fiocchi R., Cerretelli P., Skinner J.E. (1996). Heart rate variability in the human transplanted heart: Nonlinear dynamics and QT vs RR alterations during exercise suggest a return of neurocardiac regulation in long-term recovery. Integr. Psychol. Behav. Sci..

[68] Cornelissen V.A., Vanhaecke J., Aubert A.E., Fagard R.H. (2012). Heart rate variability after heart transplantation: A 10-year longitudinal follow-up study. J. Cardiol..

[69] Yamamoto Y., Nakamura Y., Sato H., Yamamoto M., Kato K., Hughson R.L. (1995). On the fractal nature of heart rate variability in humans: Effects of vagal blockade. Am. J. Physiol.-Regul. Integr. Comp. Physiol..

[70] Tumati S., Paulus M.P., Northoff G. (2021). Out-of-Step: Brain-Heart Desynchronization in Anxiety Disorders. Mol. Psychiatry.

[71] Balocchi R., Michelassi C., Varanini M., Barbi M., Chillemi S., Di Garbo A., Raimondi G., Legramante J.M. (2002). Heartbeat scaling properties in intact and denervated rabbits. WSEAS Trans. Circuits Syst..

[72] Ivanov P.C., Amaral L.A.N., Goldberger A.L., Havlin S., Rosenblum M.G., Struzik Z.R., Stanley H.E. (1999). Multifractality in human heartbeat dynamics. Nature.

[73] Frasch M.G., Herry C.L., Niu Y., Giussani D.A. (2020). First Evidence That Intrinsic Fetal Heart Rate Variability Exists and Is Affected by Hypoxic Pregnancy. J. Physiol..

[74] Finsterer J., Wahbi K. (2014). CNS-disease affecting the heart: Brain–heart disorders. J. Neurol. Sci..

[75] Young H., Benton D. (2015). We should be using nonlinear indices when relating heart-rate dynamics to cognition and mood. Sci. Rep..

[76] Thayer J.F., Lane R.D. (2007). The role of vagal function in the risk for cardiovascular disease and mortality. Biol. Psychol..

[77] Thayer J.F., Yamamoto S.S., Brosschot J.F. (2010). The relationship of autonomic imbalance, heart rate variability and cardiovascular disease risk factors. Int. J. Cardiol..

[78] Cohen B.E., Edmondson D., Kronish I.M. (2015). State of the art review: Depression, stress, anxiety, and cardiovascular disease. Am. J. Hypertens..

[79] Penninx B.W.J.H., Beekman A.T.F., Honig A., Deeg D.J.H., Schoevers R.A., van Eijk J.T.M., van Tilburg W. (2001). Depression and cardiac mortality. Arch. Gen. Psychiatry.

[80] Schneider M., Schwerdtfeger A. (2020). Autonomic dysfunction in posttraumatic stress disorder indexed by heart rate variability: A meta-analysis. Psychol. Med..

[81] Pugsley M.K., Winters B.R., Koshman Y.E., Authier S., Foley C.M., Hayes E.S., Curtis M.J. (2024). Innovative approaches to cardiovascular safety pharmacology assessment. J. Pharmacol. Toxicol. Methods.

[82] Wujtewicz M., Owczuk R. (2023). Heart rate variability in anaesthesiology—Narrative review. Anaesthesiol. Intensive Ther..

[83] Lan J.-Y., Shieh J.-S., Yeh J.-R., Fan S.-Z. (2022). Fractal Properties of Heart Rate Dynamics: A new biomarker for anesthesia—Biphasic changes in general anesthesia and decrease in spinal anesthesia. Sensors.

[84] Dinan A., de Toffol B., Pallix M., Breard G., Babuty D. (2008). Cardiac arrest: It’s all in the head. Lancet.

[85] Ryvlin P., Nashef L., Lhatoo S.D., Bateman L.M., Bird J., Bleasel A., Boon P., Crespel A., Dworetzky B.A., Høgenhaven H. (2013). Incidence and mechanisms of cardiorespiratory arrests in epilepsy monitoring units (MORTEMUS): A retrospective study. Lancet Neurol..

[86] Deisseroth K. (2015). Optogenetics: 10 years of microbial opsins in neuroscience. Nat. Neurosci..

[87] Thayer J.F., Brosschot J.F. (2005). Psychosomatics and psychopathology: Looking up and down from the brain. Psychoneuroendocrinology.

[88] Hänsel A., von Känel R. (2008). The ventro-medial prefrontal cortex: A major link between the autonomic nervous system, regulation of emotion, and stress reactivity?. BioPsychoSocial Med..

[89] Myers B. (2017). Corticolimbic regulation of cardiovascular responses to stress. Physiol. Behav..

[90] Benarroch E.E. (1993). The central autonomic network: Functional organization, dysfunction, and perspective. Mayo Clin. Proc..

[91] Forstenpointner J., Elman I., Freeman R., Borsook D. (2022). The omnipresence of autonomic modulation in health and disease. Prog. Neurobiol..

